# Diagnosis and management of patients with acute limb ischemia after Covid-19 infection: a case series

**DOI:** 10.1590/1677-5449.202200441

**Published:** 2022-11-25

**Authors:** Jamisson Garrote Teixeira, Guilherme Benjamin Brandão Pitta, Cézar Ronaldo Alves da Silva, Lucigl Regueira Teixeira, Gregório Luís Guarnieri Panazzolo, Joaquim Araújo Sampaio, Anna Karoline Rocha de Sousa, Claubiano Cipriano Moura

**Affiliations:** 1 Hospital Memorial Arthur Ramos - HMAR, Maceió, AL, Brasil.; 2 Universidade Estadual de Ciências da Saúde de Alagoas - UNCISAL, Faculdade de Medicina, Maceió, AL, Brasil.; 3 Universidade Federal de Alagoas, Hospital Universitário Professor Alberto Antunes, Maceió, AL, Brasil.

**Keywords:** Covid-19, thrombosis, anticoagulants, ischemia, lower limb, upper limb

## Abstract

The Covid-19 pandemic caused by the Sars-Cov-2 virus created challenges and stimulated development of new forms of treatment in many different areas of medicine. Studies have described the clinical characteristics of patients and their outcomes, including disorders affecting the coagulation system, in which patients infected by the virus enter a hypercoagulable and proinflammatory state that mimics vasculitis. The objective of this study was to describe the clinical status and the treatment administered to three patients who developed acute arterial occlusion after Covid-19 infection. The management adopted in these cases enabled the patients to recover without sequelae. The low incidence and scarcity of published reports make it difficult to establish universally accepted treatment criteria and options for cases of ischemia in patients infected with the novel coronavirus, whether presenting early or late.

## INTRODUCTION

The pandemic resulting from the disease caused by the Sars-Cov-2 virus (Covid-19) created challenges and stimulated development of new forms of treatment in many different areas of medicine. Studies have described the clinical characteristics of patients and their outcomes, including disorders affecting the coagulation system, in which patients infected by the virus enter a hypercoagulable and proinflammatory state that mimics vasculitis. Depending on the site of involvement, after developing inflammation of the endothelium, cases may progress to stroke, acute kidney damage, cutaneous lesions, and visceral and peripheral ischemia, in addition to ischemic states involving other organs.^1^^,^^2^

This article will describe three cases of acute limb ischemia in post-infection phases, in patients who had totally recovered from mild viral infections, but presented with proinflammatory and thrombotic sequelae. These are rare conditions, considering the very small number of cases described with this late presentation and, primarily, with benign outcomes after clinical treatment. The study was approved by the Research Ethics Committee at the Fundação Educacional Jayme de Altavila - Centro Universitário Cesmac, with Consolidated opinion number 5.166.366 and Ethics Appraisal Submission Certificate (CAAE) 53360021.1.0000.0039.

## DESCRIPTIONS OF THE CASES

### Case 1

**Figure 1:**
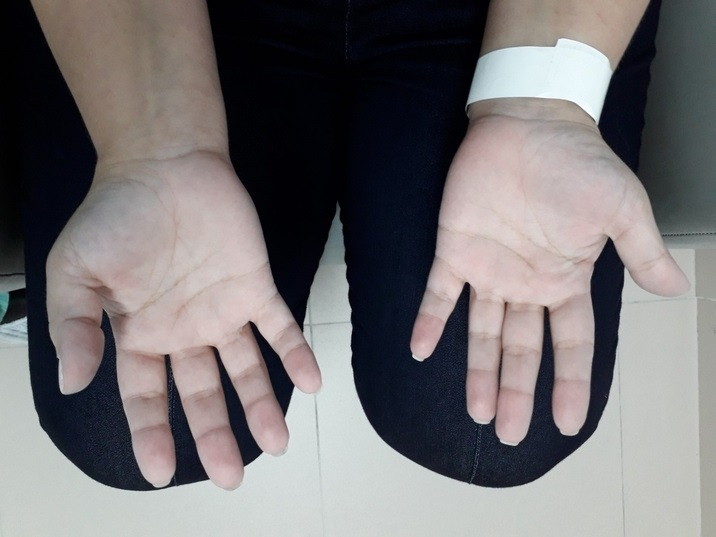
Patient presenting with reduced tissue perfusion involving the thenar area of the right hand.

**Figure 2:**
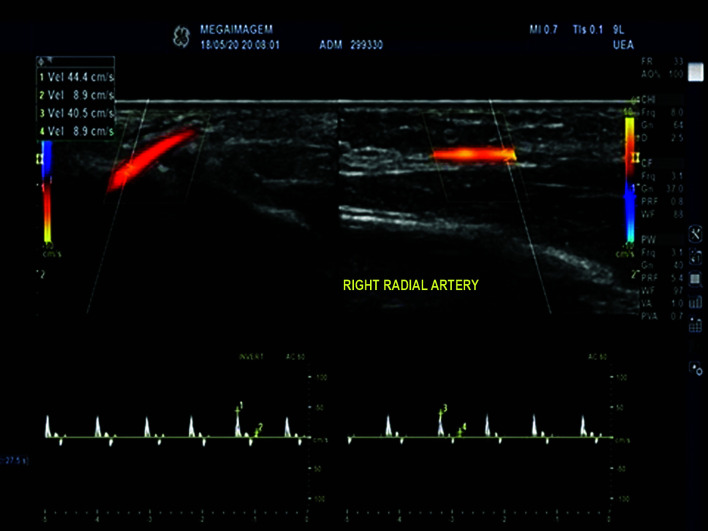
Arterial Doppler showing reduced velocity and amplitude of flow waves in the right radial artery of the wrist.

**Figure 3:**
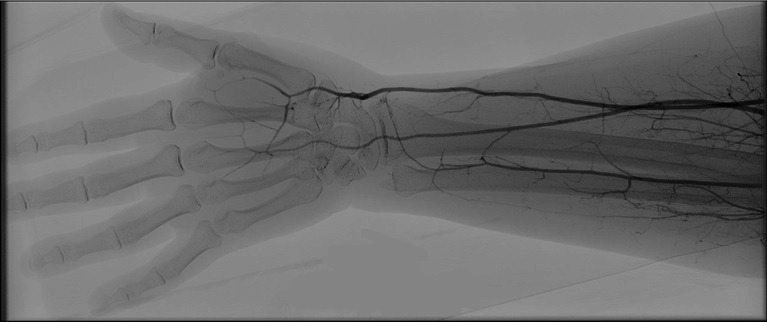
Angiography showing no opacification of the digital arteries with contrast and opacification of muscular branches, indicating obstruction of flow.

**Figure 4:**
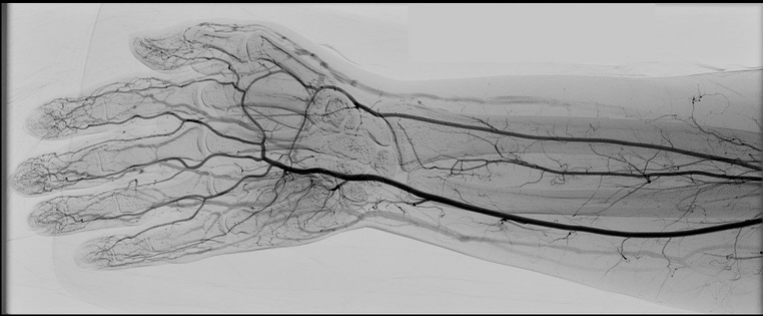
Control angiography with opacification of the digital arteries by contrast after intra-arterial heparinization.

A 27-year-old female patient presented at the emergency service with sudden onset paresthesia of the right hand and forearm, associated with cyanosis and coldness of the fingertips. On examination, her right upper limb temperature was slightly lower than the contralateral temperature, her radial pulse was present, but slightly weaker than the contralateral, and perfusion was slow in the thenar region (Figure 1). She had a history of Covid-19 infection, 30 days previously, confirmed with a positive IgG test. Table 1 lists the results of laboratory tests ordered at admission. Doppler ultrasonography was performed, showing slow blood flow through the right radial artery (Figure 2) and an absence of flow in the digital arteries. She was started on full anticoagulation with low molecular weight heparin (LMWH) and underwent arteriography of the right upper limb, which showed that the digital arteries of the right hand were not filling, contrast was being retained in the radial artery, and the ulnar artery was not filling, with evidence of contrast retention in the muscular arteries of the forearm (Figure 3). Since no failures of blood supply compatible with occlusive thrombi were observed, the decision was taken to perform intra-arterial fibrinolytic infusion and warm the limb. When checked after 6 hours, the patient reported that the paresthesia had reduced, but arteriography showed that the distal circulation had worsened. At this point, she was switched to intra-arterial unfractionated heparin (UFH), which was maintained for 24 hours, and she was started on intravenous corticoid therapy. After 24 hours under observation in an intensive care bed, the patient returned to the cath lab where angiography showed complete filling of the entire arterial network of the right upper limb (Figure 4), and her paresthesia had improved considerably. She was put back on full anticoagulation with LMWH. After 48 hours in a bed on the ward, the patient was discharged home with a prescription for LMWH (the possibility of oral direct anticoagulants was discussed with the patient, but she felt safer taking LMWH), complaining only of mild paresis and paresthesia in the hand. During outpatient follow-up, she was kept on anticoagulation for a further 45 days. She also took a 30-day course of pentoxifylline and prednisolone, prescribed by her rheumatologist, and underwent motor physiotherapy for 6 weeks, regaining normal muscle strength, although she had persistent paresthesia of the involved hand for around 12 months. At a 24-month follow-up, she had had no new ischemic episodes, paresis or paresthesia. She was vaccinated four times and was infected by the virus on two further occasions (confirmed clinically and with laboratory tests).

**Table 1:** Laboratory tests at the time of admission.

	**Patient 1**	**Patient 2**	**Patient 3**
Hemoglobin (g/dL)	12.3	12.4	11.7
Hematocrit (%)	35.8	36.6	35.7
Leukocytes/mL	8,270	6,950	9,240
Platelets/mL	269,000	158,000	363,000
D-dimer (mcg/dL)	427	530	651
CRP (mg/dL)	10.65	4.5	21.3
INR	1.22	1.21	1.0
APTTr	1.32	1.37	1.69
Urea (g/dL)	30	10	26
Creatinine (g/dL)	0.55	0.52	0.61
Glycemia (mg/dL)	109	98	74
Sodium (mg/dL)	136	135	143
Potassium (mg/dL)	3.6	3.7	4.5

INR: international normalized ratio; APTTr: activated partial thromboplastin time ratio; CRP: C-reactive protein.

### Case 2

A 44-year-old female patient presented at the emergency service with paresthesia and cyanosis of the left hand, with onset the previous day. She had a history of Covid-19 infection around 15 days earlier and was taking 40 mg of subcutaneous enoxaparin once a day. On physical examination, the patient had palpable radial and ulnar pulses bilaterally. Table 1 lists the results of laboratory tests ordered at admission. She underwent an arterial examination with Doppler ultrasound, which showed normal flow in the radial and ulnar arteries of the left upper limb, with reduced flow in the digital arteries. The patient was heparinized with intravenous UFH via an infusion pump and administered 125 mg of intravenous methylprednisolone every 12 hours and measures were taken to warm the limb passively. After 24 hours, the patient reported a considerable improvement in her paresthesia. Over the following 48 hours, she exhibited mild epistaxis and the rate of infusion of the heparin solution was reduced, with no other complications. She was discharged on the fourth day, once her symptoms had improved significantly and was maintained under ambulatory monitoring on 20 mg of rivaroxaban per day, for 45 days. At outpatient follow-up at 18 months, she had not had any further ischemic episodes. She was vaccinated four times and was infected by the virus on at least two further occasions (confirmed clinically and with laboratory tests).

### Case 3

**Figure 5:**
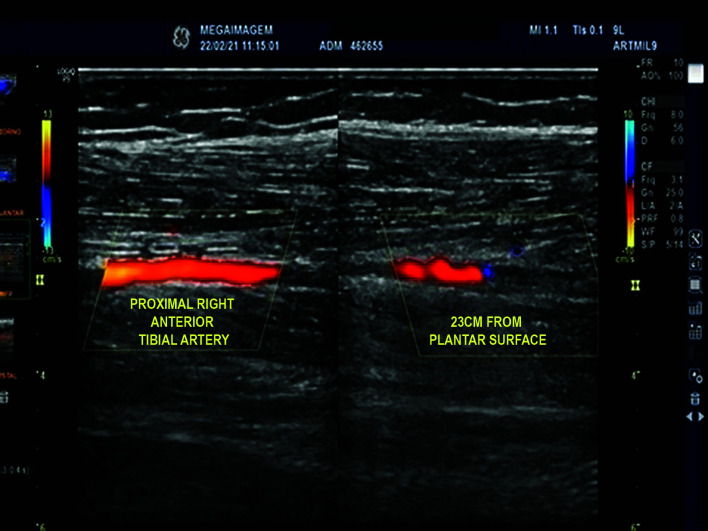
Arterial Doppler image showing occlusion of the right anterior tibial artery at 23 cm from the plantar surface.

**Figure 6:**
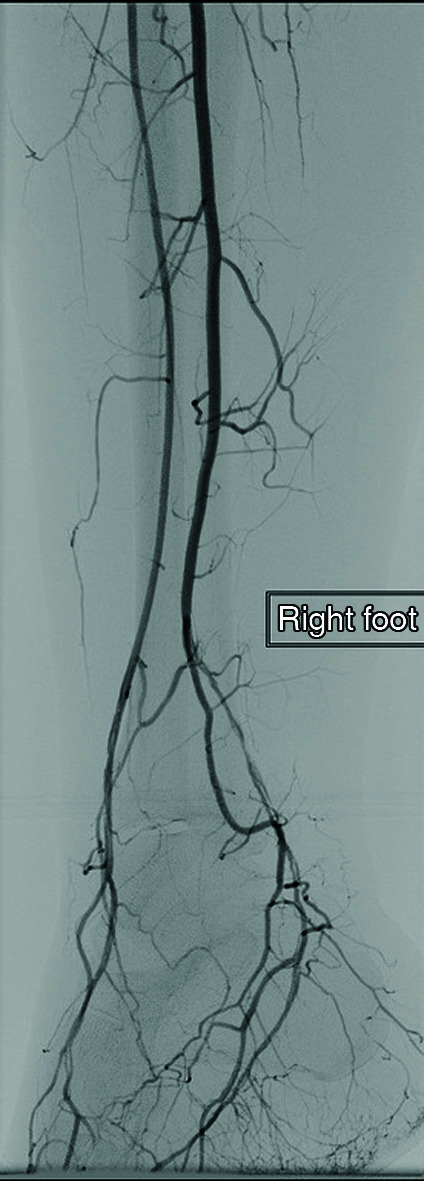
Preoperative angiography showing stenosis of the dorsalis pedis and plantaris medialis arteries (from the fibular artery, originating from the tibioperoneal trunk, which does not form the posterior tibial artery).

**Figure 7:**
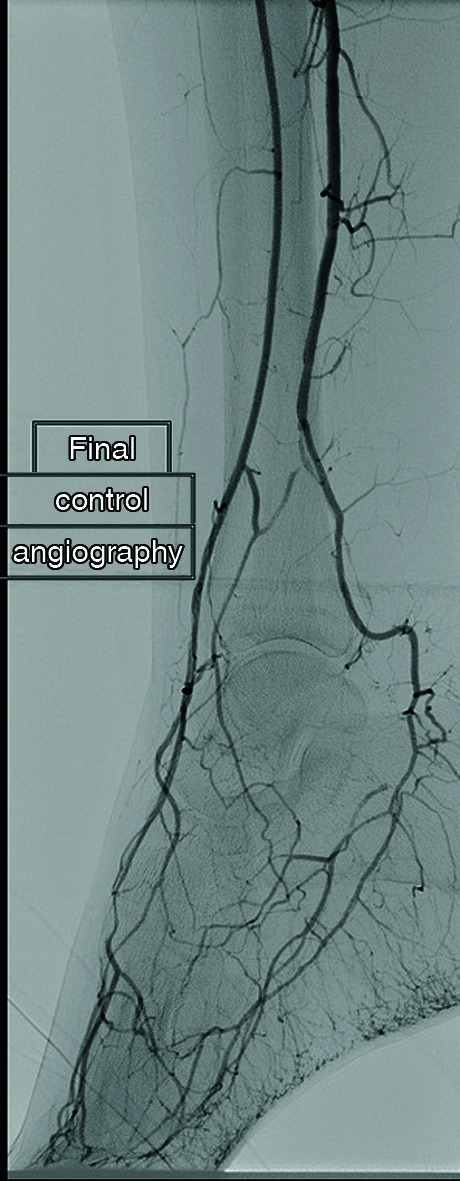
Postoperative control angiography showing improved appearance of the dorsalis pedis and plantaris medialis arteries.

A 38-year-old female patient presented at the emergency service complaining of pain in the right foot with onset about 14 days earlier, worsening progressively over the previous 24 hours, concurrently with onset of paresthesia. The patient stated that she had had a Covid-19 infection around 30 days earlier. On examination, distal pulses were absent in the right lower limb (the left pedal pulse was present) and there were signs of poor tissue perfusion in some parts of the plantar aspect and the calcaneus. She was examined with Doppler ultrasound, which revealed signs of occlusion in the tibial and dorsalis pedis arteries and slow perfusion along the plantar aspect (Figure 5). Table 1 lists the results of laboratory tests ordered at admission. Systemic heparinization was started with UFH and the limb was warmed. The patient exhibited improved local perfusion and reduced paresthesia on the fourth day and was discharged on anticoagulation with rivaroxaban. She was medicated for pain control and followed up in outpatients. She underwent a control Doppler examination 45 days later, which showed that the anterior tibial and dorsalis pedis arteries were patent and that filling of the plantar arteries was adequate, although she had tendonitis of the fibularis longus muscle, provoking localized pain along its path, and she complained of pain involving the medial and plantar aspects of the foot. Anticoagulation and outpatient follow-up were maintained. At 4 months, the patient was still symptomatic, with claudication involving the medial aspect of the foot. An exploratory angiography was scheduled, with the intention to treat if necessary. The tibioperoneal trunk was found not to have a bifurcation to the posterior tibial artery and there was stenosis of the dorsalis pedis and plantaris medialis arteries (Figure 6). Percutaneous angioplasty was performed, resulting in angiographic (Figure 7) and clinical improvement. Postoperatively, the patient’s symptomology improved, she was free from claudication and walking normally, and was kept on anticoagulation until a total of 6 months had elapsed since initial presentation. At a 15-month outpatient follow-up consultation, she had had no further ischemic episodes. The patient was vaccinated (three doses) and was infected by the virus on at least one further occasion (confirmed clinically and with laboratory testing).

## DISCUSSION

The pandemic caused by the severe acute respiratory syndrome coronavirus 2 (SARS-CoV-2) involves, in addition to the cases of alveolar pulmonary injury and acute respiratory failure, an elevated incidence of cardiovascular diseases, especially acute thrombotic events, such as arterial occlusions and venous thromboembolism (VTE),^3^ which are associated with higher mortality.^4^^,^^5^ Guarinello et al.^6^ called attention to the elevated rate of amputations among decompensated arterial patients caused by reluctance to seek medical attention for fear of contagion.^6^ From the early stages, disorders of the coagulation system in infected patients were already being reported.^7^^,^^8^^,^^9^ As knowledge about the disease was accumulated, it was suggested that the thrombotic diathesis associated with this virus is a reflection of an endothelial injury induced by infection of the endothelial cells.^10^ The thrombotic complications can be explained by this endothelial infection and induction of luminal thromboplastin (TP) expression, which acts to trigger the proteolytic cascade of thrombin and fibrin formation (extrinsic pathway or as the coagulation initiation phase).^11^^,^^12^ McGonagle et al.^1^ suggest that the virus’ mechanism of vascular involvement mimics vasculitis, which can cause cryptogenic stroke, acute kidney damage, cutaneous vasculitis, intestinal ischemia, and ischemia of other organs.^1^ A review conducted by Becker^2^ observed several different clinical presentations of vasculitis induced by SARS-CoV-2 and also a range of sites of manifestation, such as skin lesions of the toes (“covid toes”), paralysis of the trochlear nerve, and corpus callosum infarct.^2^ Alonso et al.^13^ described and quantified the incidence of acro-ischemic lesions in patients infected by Covid-19, classifying them according to their different patterns as: atypical Raynaud’s phenomenon; pseudo-pernio; severe microcirculatory ischemia; or dry gangrene with arteriosclerosis obliterans.^13^ Pulmonary embolism was the most common thrombotic event among these patients, despite administration of thromboprophylaxis in hospital in some cases.^14^ Reports have been published describing different clinical presentations and management of these events, such as ischemic stroke,^15^ deep venous thrombosis (DVT), mesenteric ischemia,^16^ thrombosis of the abdominal aorta and common iliac artery bilaterally,^17^ arterial thrombosis limbs in isolation,^18^^,^^19^ and even association with autoimmune diseases.^20^ Teng et al.^21^ described the case of a patient who had two episodes of acute arterial occlusion of the right lower limb during the infection and even using anticoagulant therapy (due to the patient having developed pulmonary embolism).^21^ Management of arterial events includes surgical treatment with thromboembolectomy, supplemented or not by adjuvant therapy with intra-arterial fibrinolytics and/or systemic heparinization.^22^ Rosa et al.^23^ described cases of upper limb ischemia diagnosed during acute presentation of SARS-CoV-2 in which a combination of heparin and corticoid with alprostadil was used, with varying outcomes depending on the severity of the cases.^23^ That article also differs from our study in terms of the patients’ D-dimer values at admission, which may be related to the lower severity and earlier diagnoses in the cases we treated. The cases described by Rosa et al.^23^ are notable for their late presentation, which, after knowledge was accumulated during the first case seen at the service, proved to be possible to diagnose noninvasively, with purely clinical management or clinical management followed up with endovascular treatment later. In the first two cases, we describe arterial events during the post-infection period and in patients who had had mild cases of viral infection, which differs from the episodes of arterial thromboembolism described in the literature. In cases 1 and 2, we describe patients with early symptoms, which were probably identified during the initial distal vasospasm phase of the virus-induced vasculitis (or because of the highly inflammatory state that these patients may be in) and were treated before they progressed to formation of arterial thrombi. In case 1, because of the length of time during which the hand remained hypoperfused, there was paresthesia and paresis, which were both reversible. In case 2, treatment was promptly started with a corticoid (low dose) and systemic heparinization because of the experience with, and the result of, case 1 (the first post-Covid-19 case seen at our service). In contrast, in case 3, the patient sought care at a later point, when thrombotic involvement had already occurred. In this case, the same clinical treatment was chosen, in order not to conduct a procedure immediately to avoid the complications of an inflammatory process. The patient responded well to treatment with intravenous heparinization, and the condition was treated later with angioplasty. It is important to highlight the value of vascular echography for diagnosis of the condition. This is a noninvasive examination and since it is extremely accurate when performed by experienced examiners, it is capable of guiding intrahospital treatment and can even be used for planning later surgery. Both patient 2 and patient 3 were diagnosed and had treatment defined using this examination alone, without a need for invasive methods or examinations with contrast. Thus, episodes of acute occlusions secondary to infection with the SARS-CoV-2 virus can occur both in severe patients during the infection or at a later date after the viral presentation, even if the infection has been mild, and can even be treated in a less invasive manner, providing that they are identified early. These findings are different from those found, for example, by Galyfos et al.,^24^ who stated that mortality was higher among cases treated conservatively.^24^ At the time of diagnosis of respiratory infection, only patient 2 was prescribed enoxaparin, even though it was a mild case, and none of the patients were diagnosed with thrombophilia during ambulatory investigations and care, inferring that the events were exclusively triggered by the virus-induced vasculitis.^25^^,^^26^

## CONCLUSIONS

Although the spread has slowed down, there is still an elevated global incidence of SARS-CoV-2 infection and new strains are emerging, maintaining the relationship with thrombotic events and the importance of dealing with the issue. The virus appears to establish a pro-thrombotic state by means of endotheliitis and while there is greater risk of thrombotic events in serious cases, the risk is not absent in mild cases, even after a period of convalescence from the respiratory disease. The low incidence and the small number of published reports make it difficult to establish universally accepted treatment criteria and options for cases of ischemia in patients infected by the novel coronavirus. In both early and late presentations, a high degree of suspicion should be maintained to achieve diagnosis and initiate treatment as early as possible. We present our experience, in which, in cases that manifested late, patients were treated, there was no recurrence of ischemic episodes, and they did not suffer sequelae.
