# Supplementary material for: Diagnosis and management of patients with acute limb ischemia after Covid-19 infection: a case series
Source: J Vasc Bras. 2022 Nov 25;21:e20220044. doi: 10.1590/1677-5449.202200441 (PMC9716358; doi:10.1590/1677-5449.202200441)
Supplement: Translation

# Translation: Diagnóstico e manejo de isquemia aguda de membro em paciente pós infecção viral por covid-19: série de casos

**DOI:** 10.1590/1677-5449.202200442

**Authors:** Jamisson Garrote Teixeira, Guilherme Benjamin Brandão Pitta, Cézar Ronaldo Alves da Silva, Lucigl Regueira Teixeira, Gregório Luís Guarnieri Panazzolo, Joaquim Araújo Sampaio, Anna Karoline Rocha de Sousa, Claubiano Cipriano Moura

**Affiliations:** 1 Hospital Memorial Arthur Ramos - HMAR, Maceió, AL, Brasil.; 2 Universidade Estadual de Ciências da Saúde de Alagoas - UNCISAL, Faculdade de Medicina, Maceió, AL, Brasil.; 3 Universidade Federal de Alagoas, Hospital Universitário Professor Alberto Antunes, Maceió, AL, Brasil.

**Keywords:** covid-19, trombose, anticoagulantes, isquemia, extremidade inferior, extremidade superior

## Abstract

A pandemia causada pela doença do coronavírus 2019 (covid-19) estabeleceu desafios e estimulou o desenvolvimento de diferentes formas terapêuticas em várias áreas médicas. Estudos descreveram características clínicas e desfechos de pacientes, incluindo desordens do sistema de coagulação, em que os pacientes infectados pelo vírus apresentam um estado de hipercoagulabilidade e pró-inflamatório que mimetiza uma vasculite. O objetivo deste trabalho foi descrever o quadro clínico e a terapia empregada em uma série de três pacientes que evoluíram com quadro de oclusão arterial aguda no período pós-infecção por covid-19. O manejo aplicado nesses casos permitiu que os pacientes evoluíssem sem sequelas. A baixa incidência e a pobreza em relatos descritos tornam difícil o estabelecimento de critérios e opções terapêuticas universalmente aceitos nos casos de isquemia, seja precoce ou tardia, dos pacientes infectados pelo novo coronavírus.

## INTRODUÇÃO

A pandemia ocorrida pela doença do coronavírus 2019 (covid-19) estabeleceu desafios e estimulou o desenvolvimento de diferentes formas terapêuticas em várias áreas médicas. Diferentes estudos descreveram variadas características clínicas e desfechos de pacientes, incluindo desordens do sistema de coagulação, em que os pacientes infectados pelo vírus apresentam um estado de hipercoagulabilidade e pró-inflamatório que mimetiza uma vasculite. A partir do desenvolvimento da inflamação do endotélio, o paciente pode evoluir, de acordo com o local de acometimento, com acidente vascular encefálico, lesão renal aguda, lesões cutâneas e isquemias viscerais e periféricas, além de quadros isquêmicos em outros órgãos^1^^,^^2^.

Serão descritos três casos de isquemia aguda de membros em estágio pós-infecção, com pacientes totalmente recuperados de um quadro viral brando, porém apresentando sequelas do seu *status* pró-inflamatório e trombótico. Trata-se de uma condição rara, tendo em vista pouquíssimos casos descritos com essa apresentação tardia e principalmente com evolução benigna através de tratamento clínico. O estudo foi aprovado pelo comitê de ética em pesquisa da Fundação Educacional Jayme de Altavila - Centro Universitário Cesmac, sob Parecer Consubstanciado de número 5.166.366 e Certificado de Apresentação de Apreciação Ética (CAAE) 53360021.1.0000.0039.

## DESCRIÇÃO DOS CASOS

### Caso 1

Uma paciente do sexo feminino, de 27 anos, deu entrada no serviço de emergência com quadro de parestesia em mão e antebraço direitos, de início súbito, associada a cianose e sensação de frio nas pontas dos dedos. Ao exame, apresentava membro superior direito com temperatura discretamente inferior ao contralateral, pulso radial presente pouco diminuído em relação ao contralateral, perfusão lentificada em região tenar (Figura 1). A paciente tinha história pregressa de infecção por covid-19 30 dias antes, já confirmada com IgG positivo. Exames laboratoriais de admissão podem ser visualizados na Tabela 1. Foi solicitada ultrassonografia Doppler, que evidenciou fluxo lentificado em artéria radial direita (Figura 2) e ausência de fluxo nas artérias digitais. Foi iniciada anticoagulação plena com heparina de baixo peso molecular (HBPM), e a paciente foi submetida a arteriografia do membro superior direito, que evidenciou ausência de enchimento das artérias digitais da mão direita, retenção do contraste na artéria radial e ausência de enchimento da artéria ulnar, com retenção e evidência das artérias musculares do antebraço (Figura 3). Como não foi evidenciada nenhuma falha de enchimento compatível com trombo oclusivo, optou-se pela realização de infusão de fibrinolítico intra-arterial, além do aquecimento do membro. No controle 6 horas após, a paciente referia diminuição da parestesia, e a arteriografia demonstrou piora do aspecto da circulação distal. Nesse momento, foi introduzida terapia com heparina não fracionada (HNF) intra-arterial, mantida por 24 horas, e foi iniciada terapia com corticoide endovenoso. Após período de 24 horas em observação em leito do Centro de Terapia Intensiva (CTI), a paciente retornou para a hemodinâmica, e a angiografia mostrou enchimento completo de toda rede arterial do membro superior direito (Figura 4), evidenciando melhora importante da parestesia. Optou-se por substituição para anticoagulação plena com HBPM. Após 48 horas em leito de enfermaria, a paciente recebeu alta mantendo uso domiciliar de HBPM (foi discutido com paciente a possibilidade de anticoagulantes orais diretos, porém ela sentiu-se mais segura com uso de HBPM), queixando-se somente de parestesia e paresia leve na mão. No acompanhamento ambulatorial, foi mantida a anticoagulação por mais 45 dias. A paciente fez uso por 30 dias de pentoxifilina e prednisolona a pedido de seu reumatologista. Realizou ainda fisioterapia motora por 6 semanas, retornando a força muscular ao normal, e manteve parestesia na mão acometida por cerca de 12 meses. No seguimento de 24 meses, não houve novos episódios isquêmicos, paresias ou parestesias; a paciente foi submetida a vacinação (quatro doses), tendo sido infectada outras duas vezes pelo vírus (clínica e laboratorialmente confirmada).

**Figura 1:** Paciente apresentando perfusão tecidual diminuída em região tenar da mão direita.

**Tabela 1:** Exames laboratoriais no momento da admissão.

	**Paciente 1**	**Paciente 2**	**Paciente 3**
Hemoglobina (g/dL)	12,3	12,4	11,7
Hematócrito (%)	35,8	36,6	35,7
Leucócitos/mL	8.270	6.950	9.240
Plaquetas/mL	269.000	158.000	363.000
D-dímero (mcg/dL)	427	530	651
PCR (mg/dL)	10,65	4,5	21,3
RNI	1,22	1,21	1,0
rTTPA	1,32	1,37	1,69
Ureia (g/dL)	30	10	26
Creatinina (g/dL)	0,55	0,52	0,61
Glicemia (mg/dL)	109	98	74
Sódio (mg/dL)	136	135	143
Potássio (mg/dL)	3,6	3,7	4,5

RNI: razão de normatização internacional; rTTPA: relação de tempo de tromboplastina parcial ativada; PCR: proteína C-reativa.

**Figura 2:** Doppler arterial apresentando diminuição de velocidade e amplitude das ondas do fluxo da artéria radial direita no punho.

**Figura 3:** Angiografia apresentando não opacificação de artérias digitais pelo contraste e opacificação de ramos musculares, denotando represamento do fluxo.

**Figura 4:** Controle angiográfico com opacificação das artérias digitais pelo contraste após heparinização intra-arterial.

### Caso 2

Uma paciente do sexo feminino, de 44 anos, deu entrada no setor de emergência devido à parestesia e cianose em mão esquerda de início há 1 dia. Havia história prévia de infecção por covid-19 há cerca de 15 dias, e ela estava em uso de enoxaparina 40 mg subcutâneo uma vez ao dia. Ao exame físico, a paciente apresentava pulsos radial e ulnar palpáveis bilateralmente. Exames laboratoriais de admissão podem ser visualizados na Tabela 1. Ela foi submetida a exame ultrassom Doppler arterial, que evidenciava fluxo normal em artérias radial e ulnar no membro superior esquerdo, com captação de fluxo reduzida nas artérias digitais. Optou-se pela heparinização com HNF endovenosa em bomba de infusão, metilprednisolona 125 mg de 12 em 12 horas endovenosa e medidas para aquecimento passivo do membro. Após 24 horas, a paciente referia redução importante da parestesia. No período de 48 horas, a paciente apresentou discreta epistaxe, sendo diminuída a taxa de infusão da solução de heparina, sem outras intercorrências. A paciente recebeu alta no quarto dia após melhora importante dos sintomas, sendo acompanhada ambulatorialmente com uso de rivaroxabana 20 mg por dia, que foi mantido por 45 dias. No acompanhamento ambulatorial de 18 meses, não apresentou novos episódios isquêmicos; a paciente foi submetida a vacinação (4 doses), tendo sido infectada pelo menos duas vezes pelo vírus (clínica e laboratorialmente confirmadas).

### Caso 3

Uma paciente do sexo feminino, de 38 anos, deu entrada no setor de emergência com relato de dor em pé direito há cerca de 14 dias, com piora progressiva nas últimas 24 horas associada a parestesia. A paciente relatou infecção por covid-19 há cerca de 30 dias. Ao exame, apresentava, em membro inferior direito, ausência de pulsos distais (pulso pedioso esquerdo presente) e sinais de má perfusão tecidual em algumas áreas da face plantar e calcâneo. Ela foi submetida a exame ultrassom Doppler, que evidenciou sinais de oclusão em artérias tibiais e dorsal do pé e lentificação da perfusão em face plantar (Figura 5). Exames laboratoriais de admissão podem ser visualizados na Tabela 1. Optou-se por início de heparinização sistêmica com HNF e aquecimento do membro. A paciente apresentou melhora da perfusão local e da parestesia no quarto dia, recebendo alta com anticoagulação com rivaroxabana; ela foi medicada para controle da dor, e foi mantido acompanhamento ambulatorial. Paciente realizou Doppler de controle 45 dias após, que evidenciou artérias tibial anterior e dorsal do pé pérvias e enchimento adequado das artérias plantares, tendinite de músculo fibular longo, cursando com dor local em sua topografia, queixando-se de dor em face medial e plantar do pé. Foram mantidos anticoagulação e seguimento ambulatorial. Após 4 meses, a paciente manteve-se sintomática, com claudicação em face medial do pé. Foi indicada angiografia armada, evidenciando tronco tibiofibular sem bifurcação para artéria tibial posterior e estenoses em artéria pediosa e plantar medial (Figura 6), sendo realizada angioplastia percutânea com melhora angiográfica (Figura 7) e clínica. A paciente evoluiu no pós-operatório com melhora da sintomatologia, sem claudicação, deambulando normalmente, sendo mantida anticoagulação até completar 6 meses do quadro inicial. No acompanhamento ambulatorial de 15 meses, não houve novos episódios isquêmicos; a paciente foi submetida a vacinação (3 doses), tendo sido infectada pelo menos mais uma vez pelo vírus (clínica e laboratorialmente confirmada).

**Figura 5:** Doppler arterial evidenciando oclusão da artéria tibial anterior direita a 23 cm da face plantar.

**Figura 6:** Angiografia pré-operatória evidenciando estenoses em artéria dorsal do pé e plantar medial (originária da artéria fibular, advinda de tronco tibiofibular que não forma artéria tibial posterior).

**Figura 7:** Angiografia pós-operatória de controle evidenciando melhora do aspecto das artérias dorsal do pé e plantar medial.

## DISCUSSÃO

A pandemia causada pelo coronavírus 2 da síndrome respiratória aguda grave (SARS-CoV-2) apresenta, além de casos de lesão pulmonar alveolar e falência respiratória aguda, elevada incidência de doenças cardiovasculares, em especial eventos trombóticos agudos, como oclusões arteriais e tromboembolismo venoso (TEV)^3^, que estão relacionados com uma maior mortalidade^4^^,^^5^. Guarinello et al.^6^ chamaram atenção para o elevado índice de amputações em pacientes arteriais descompensados devido ao receio de contágio ao buscar atendimento médico^6^. Ainda em seu início, desordens do sistema de coagulação foram citadas nos pacientes infectados^7^^,^^8^^,^^9^. Com o avançar do conhecimento sobre a doença, foi sugerido que a diátese trombótica associada ao vírus reflete uma endoteliopatia induzida pela infecção das células endoteliais^10^. As complicações trombóticas podem ser explicadas por essa infecção endotelial e a indução da expressão luminal do fator tecidual (FT), que age como um gatilho da cascata proteolítica de formação de trombina e fibrina (via extrínseca ou fase de iniciação da coagulação)^11^^,^^12^. McGonagle et al.^1^ sugerem que o mecanismo de acometimento vascular do vírus seria mimetizando uma vasculite, que poderia levar a acidente vascular cerebral criptogênico, lesão renal aguda e vasculites cutâneas, isquemia intestinal e outras isquemias de outros órgãos^1^. Um levantamento realizado por Becker^2^ observou várias apresentações clínicas da vasculite induzida pelo SARS-CoV-2, assim como diferentes locais de manifestação, como lesões cutâneas nos dedos do pé (“*covid toes*”), paralisia de nervo troclear e infarto de corpo caloso^2^. Alonso et al.^13^ descreveram e quantificaram a incidência de lesões acrais isquêmicas (LAI) em pacientes infectados por covid-19, agrupando-as em diferentes padrões: fenômeno de Raynauld atípico; pseudopernio; isquemia grave da microcirculação; e gangrena seca com *arteriosclerosis obliterans*^13^. A embolia pulmonar foi identificada como o evento trombótico mais comum nesses pacientes, apesar da aplicação de tromboprofilaxia durante o internamento em alguns casos^14^. Já foram publicados relatos de diferentes apresentações clínicas e condutas frente a esses eventos, como acidente vascular encefálico isquêmico (AVEi)^15^, trombose venosa profunda (TVP), isquemia mesentérica^16^, trombose de aorta abdominal e de artéria ilíaca comum bilateralmente^17^, trombose arterial de membros isoladamente^18^^,^^19^ e até mesmo associação com doenças autoimunes^20^. Teng et al.^21^ descreveram o caso de um paciente que, durante a infecção e mesmo em uso de terapia anticoagulante (pois o paciente havia desenvolvido embolia pulmonar), apresentou dois episódios de oclusão arterial aguda em membro inferior direito^21^. Entre os eventos arteriais, as condutas têm sido relatadas com a terapêutica cirúrgica pela tromboembolectomia, complementada ou não por terapia adjuvante com fibrinolítico intra-arterial e/ou heparinização sistêmica^22^. Rosa et al.^23^ descreveram casos de isquemia de membros superiores diagnosticados na apresentação aguda do SARS-CoV-2, em que se utilizou associação de heparina e corticoide com alprostadil, tendo desfechos variados relacionados com a gravidade dos pacientes^23^. Esse estudo também difere do nosso quanto aos valores de D-dímero na admissão dos pacientes, podendo estar relacionado com a menor severidade e os diagnósticos precoces dos casos do presente artigo. Os casos descritos no estudo de Rosa et al.^23^ chamam atenção pela apresentação tardia, que, após conhecimentos adquiridos com o primeiro caso do serviço, mostraram-se passíveis de diagnóstico não invasivo, com manejo puramente clínico ou associado a tratamento endovascular em segundo tempo. Nos dois primeiros casos, apresentamos eventos arteriais no período pós-infecção e em pacientes que passaram por quadros leves de infecção viral, o que difere dos episódios relatados na literatura quanto a tromboembolismo arterial. Nos casos 1 e 2, relatamos pacientes com sintomas precoces, tendo sido identificadas provavelmente no período inicial de vasoespasmo distal do quadro da vasculite induzida pelo vírus (ou pelo estado altamente inflamatório em que os pacientes podem se encontrar) e tratadas antes da evolução para formação de trombos arteriais. O caso 1, pelo tempo que a mão esteve hipoperfundida, apresentou parestesia e paresia, as quais foram reversíveis. No caso 2, foi iniciada precocemente a terapia com corticoide (dose baixa) e heparinização sistêmica devido à experiência e ao resultado do caso 1 (primeiro caso pós-covid-19 atendido em nosso serviço). O caso 3 já mostra uma paciente que buscou atendimento em momento mais tardio, quando já havia ocorrido comprometimento trombótico. Nesse caso, decidiu-se pelo mesmo tratamento clínico, para não realizar procedimento naquele momento em busca de evitar as complicações do processo inflamatório. A paciente apresentou boa resposta à terapia com heparinização endovenosa, tendo o quadro por fim tratado posteriormente com angioplastia. É importante salientar o valor da ecografia vascular no diagnóstico do quadro. Por ser um exame não invasivo e extremamente acurado quando realizado por examinadores experientes, ela mostrou-se capaz de nortear o tratamento intra-hospitalar e mesmo a programação cirúrgica em momento posterior. As pacientes 2 e 3 tiveram diagnóstico e terapêutica definidos somente com o exame, sem uso de métodos invasivos ou contrastados. Logo, episódios de oclusões agudas por infecção do vírus SARS-CoV-2 podem ocorrer tanto em pacientes graves na vigência de infecção como em período tardio após o quadro viral, mesmo que tenha sido leve, que pode até mesmo ser tratado de forma menos invasiva, desde que identificado precocemente. Esses achados são diferentes dos encontrados, por exemplo, por Galyfos et al.^24^, que afirmam haver uma maior mortalidade nos casos tratados de forma conservadora^24^. No momento do diagnóstico da infecção respiratória, apenas a paciente 2 recebeu prescrição de enoxaparina, mesmo tratando-se de caso leve, e nenhuma foi diagnosticada com trombofilia durante o acompanhamento e a investigação ambulatorial, inferindo que os eventos foram desencadeados exclusivamente pela vasculite induzida pelo vírus^25^^,^^26^.

## CONCLUSÕES

Apesar de um ritmo menor, ainda há elevada incidência global de infecção pelo SARS-CoV-2, bem como surgimento de novas cepas, mantendo sua relação com eventos trombóticos e a importância em tratar do tema. O vírus parece estabelecer um *status* pró-trombótico por meio de uma endotelite, havendo um maior risco de eventos trombóticos nos pacientes graves, porém não ausente em casos leves, mesmo após período de convalescença da doença respiratória. A baixa incidência e o pequeno número de relatos descritos tornam difícil o estabelecimento de critérios e opções terapêuticas universalmente aceitas nos casos de isquemia nos pacientes infectados pelo novo coronavírus. Tanto nas apresentações precoces quanto nas tardias deve-se agir com alta suspeição para obter um diagnóstico e introduzir o tratamento o mais precocemente possível. Expusemos nossa experiência, em que, nos casos citados e manifestados tardiamente, as pacientes foram tratadas, não houve recorrência de episódios isquêmicos e elas não apresentam sequelas.
